# A Caregiver Support Platform within the Scope of an Ambient Assisted Living Ecosystem

**DOI:** 10.3390/s140305654

**Published:** 2014-03-20

**Authors:** Angelo Costa, Paulo Novais, Ricardo Simoes

**Affiliations:** 1 CCTC-Computer Science and Technology Center, University of Minho, Braga 4710-057, Portugal; E-Mail: acosta@di.uminho.pt; 2 Institute for Polymers and Composites—IPC/I3N, University of Minho, Campus de Azurém, Guimarães 4800-058, Portugal; E-Mail: rsimoes@dep.uminho.pt; 3 Life and Health Sciences Research Institute (ICVS), School of Health Sciences, University of Minho, Campus de Gualtar, Braga 4710-057, Portugal; 4 Polytechnic Institute of Cavado and Ave, Campus do IPCA, Barcelos 4750-810, Portugal

**Keywords:** ambient assisted living, ambient intelligence, intelligent environments, AAL4ALL, e-health, active ageing, artificial intelligence

## Abstract

The Ambient Assisted Living (AAL) area is in constant evolution, providing new technologies to users and enhancing the level of security and comfort that is ensured by house platforms. The Ambient Assisted Living for All (AAL4ALL) project aims to develop a new AAL concept, supported on a unified ecosystem and certification process that enables a heterogeneous environment. The concepts of Intelligent Environments, Ambient Intelligence, and the foundations of the Ambient Assisted Living are all presented in the framework of this project. In this work, we consider a specific platform developed in the scope of AAL4ALL, called UserAccess. The architecture of the platform and its role within the overall AAL4ALL concept, the implementation of the platform, and the available interfaces are presented. In addition, its feasibility is validated through a series of tests.

## 1. Introduction

Modern civilization is living on the brink of technological innovation. Never before have technological products evolved as much as in the last 15 to 20 years. One of the reasons for this evolution leap was the introduction of consumer electronics, which allowed the common population to have easy access to advanced electronic devices. Nowadays, most people are used to owning and operating advanced systems [1]. Thus, society in general has taken on technological devices as an absolute common good, providing a shift in the way that *electronic and digital tools* are being used. For instance, we can observe the way that people use computers and smartphones, which have expanded beyond their initial purpose of work facilitators and communication devices to become complete and complex entertainment systems with games, music, and videos.

Still another driving force in the technological area were evolutions in other domains which were only imminently technological, such as the medical field, engineering practice, and telecommunications. They require massive investments, which led to cutting-edge technology solutions that are used to solve complex problems [2–4]. Moreover, even the relatively minor developments played an important role, by inducing a technological development mentality that has shaped the world we know, and which is continuously and steadily progressing.

We have to recognize society's contribution in stimulating the advance of technology. It was the acceptance and the subsequent demand of the population that allowed a very rapid growth of this sector. Yet another aspect that emerged from this demand were user-centred devices, which led to the realization that simple appliances would have to adjust to the user, rather the user having to adjust to the appliances. A specific and obvious example is home domotics.

Home domotics had a fairly humble start, with the semi-automation of simple actions, such as motorized windows blinds, which require human interaction to operate. Its evolution naturally gave rise to the bypass of the user intervention in the automation process, which picking the previous example, meant fully automated windows blinds that automatically adjust their status according to weather, light and temperature conditions [5,6]. But there is a fundamental problem with this system: its cost/effectiveness ratio; thus, “old” systems are still being mounted in new homes. Another problem is the real integration of domotics. The previously referred technology evolution still has not yet had a significant repercussion in domotics. Meaning, there is an eerie lack of integration of devices and services at the home environment, although laboratory-scale projects and a few practical implementations have proven the practicability of the integration of heterogeneous systems, a domain termed Intelligent Environments.

Intelligent Environments (IEs) aim at the development of technological environments that allow communication between every device, whether sensors or actuators, while at the same time retrieving the context for each environment's state [7]. In [8] a few advances were presented that allowed the construction of IEs, namely:

- Device miniaturization; the small form factors of hardware allowed devices such as modern smartphones and intelligent pills that record several vital signs and information of a patient [9].
- The large quantity of information available derived from a multitude of sources (e.g., cameras, thermometers, Wi-Fi networks, shopping profiles, weather conditions, among others), the classification of said information (whether manually or automatically), and the generation of knowledge (by data fusion, action prediction, and environment identification) [10].
- The exponential increase of computing power and processor architecture optimization, along with the decrease in power consumption. Hardware, such as processors, is now breaking barriers faster than ever before and we are witnessing the advent of specialized hardware for certain tasks that produce considerably better results than generic ones.
- The rapid growth of the Web of Things, which leads to the integration of advanced features in even the most common devices, creating ubiquitous systems and allowing the use of high-level information trading, thus generating complex context information of the environment's events. To support the context information, new software platforms were developed with the ability to process heterogeneous information [11].
- Adaptive user interfaces and user profile detection, allowing personalized information display and the automatic and seamless adaptation to different user constraints.
- Intelligent functions (such as learning and reasoning), that allow the environment to consider the specific user (by detecting emotions, movements and actions), and adapt itself to those events.

Therefore, IEs can be perceived as a large umbrella that encompasses the Ambient Intelligence (AmI) and the Ambient Assisted Living (AAL) areas, which are the main themes of this work. The UserAccess project is presented along with state of the art projects in the previously mentioned areas.

### AmI in the AAL Context

AmI is a fast growing area that aims at the implementation of high level functionality enhancing the behaviour of environments [12–14]. To this end, environments are imbued with the ability of obtaining not only data but assigning meaning to it, thus establishing a context. An important feature is the layering of contexts, meaning that there is the ability of creating alliances of different devices (in the broadest sense of sensors and actuators), with the goal of managing less complex actions, controlling the middleware, and creating networks that trade simpler but richer messages. In practical terms, the implemented system does a real-time analysis of the environment, monitoring events, and providing an adjusted and timely response which enables it to interact with the environment's inhabitants.

**Figure 1.**
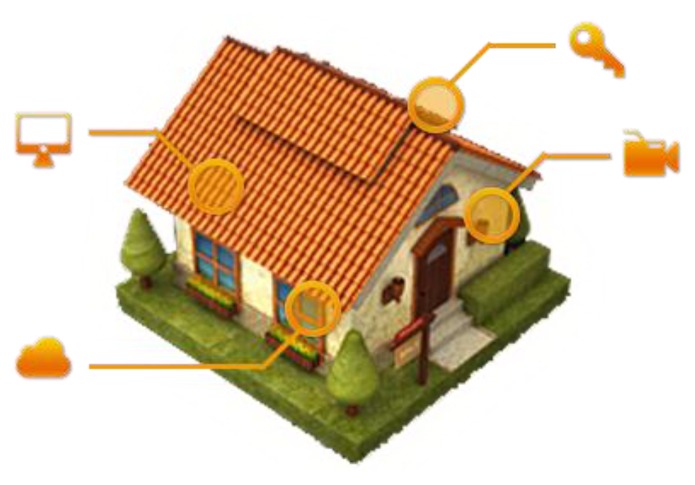
Integrated services in an AmI home environment. The integration process is responsible for the homogenization of heterogeneous systems, such as flood sensors with video capture.

Therefore, AmI stands as a true enhancement of domotics, as illustrated in Figure 1. Not only does it provide efficiency to any environment, but it establishes a central processing unit able to respond more intelligently to the environment's conditions. A typical setting for an AmI environment is a house. The house should be equipped with different sensor systems that connect to a central system, able to sort the incoming information and determine compound events, which relate to user actions. Another property of the AmI systems is the ability to choose the maximizing feature. For instance, the system economy profile is different from the comfort profile. There can be different profiles, but, due to the possible concurrency, there can be only one active at a certain time, and thus, maximization can be achieved. The following scenario is representative of a user action and the AmI system response.

**Scenario 1:** a home is located in a region that has an average outside temperatures of over 40 degrees Celsius in the summer and 10 or less degrees Celsius in the winter. The house is equipped with an air-conditioning system (AC), motorized windows blinds, and indoor and outdoor thermal sensors, as well as a set of diverse actuators and smart appliances. If the objective is to save energy, the AmI system can sort the combination of blinds positioning, aiming for the least usage of the AC. Whilst, if the objective is comfort, the system uses the blinds to control the light intensity and not the temperature, leaving this task to the AC.

As showed in Scenario 1, the maximization objective restrains the system actions and decisions. The term “decisions” is used loosely as in the standard AmI systems they are only lightly proactive, with most of the system's actions being the outcome of reactive programming. The reactive system provides fast response times and is very reliable, being used in most security procedures, although it lacks the ability to respond to new events and unplanned scenarios. One of the particularities that was introduced by AmI systems are the communication protocols, allowing the reception of high-level and low-level data and combining them to achieve the desired results.

The level of complexity (and thus, context awareness, and intelligent response) that can be introduced can grow exponentially by simply adding more sensors, actuators or even personalized user preferences. Thus, AmI platforms are designed to support those levels of complexity and to be flexible enough to allow new additions or be able to change present configurations to better suit the inhabitants' requirements.

One can say that there are also social motivations behind the AmI concept. Recent projects tend to have a central motivation on social issues, and are aimed at specific sectors of the population, such as the elderly and those with cognitive or physical impairments. The several scopes that AmI can operate in, such as security, wellbeing, safety, health, and entertainment, can be directly used to increase the user's autonomy and safety [15]. This specific domain is the responsibility of AAL.

AAL attends to a specific population that has unique constraints and require personalized technological solutions. Commonly associated to the elderly population, AAL is in fact concerned with population that have a variety of limitations or impairments, irrespective of age. The difference is that recent studies [16–19] have shown that the elderly population is rapidly increasing, surpassing the combined number of teenagers and children in advanced countries. In addition, elderly consistently exhibit some restrictions that appear naturally along the years. This means that there is currently already a high demand of solutions that can assist the elderly population, reflected in the aim of contemporary projects. It is also important to consider that, given current demographic trends, the cost associated with some of the traditional assistance needs of elderly will clearly soon become unrealistic and advanced technological solutions must emerge, which are capable of delivering even better care while simultaneously reducing current costs.

Being complementary to AmI, the AAL area reflects similar advances, and contributes with innovative solutions that can reach the general population. AAL is set on two concepts: security and comfort [20–26]. These two concepts are very broad and portrait different perspectives. For instance, the security concept can mean protecting the user against external threats (such as burglars or natural catastrophes), but also protecting users from themselves (such as monitoring falls or identifying actions that may cause physical or psychological damage). On the other hand, comfort means providing the best possible environment to the inhabitant, caring for their wellbeing, entertainment, daily tasks and general events. Therefore, there is an important difference between AmI and AAL: the concept of environment.

The following persona (the outcome of a cluster that gathers the main features that the elderly possess) is a typical user of an AAL environment.

**Persona 1:** Maria lives alone in a good house. She attended and graduated primary school. She currently lives on her monthly retirement allowance of about 300€. She sometimes feels lonely but lacks the will and motivation to have a more active social life. She is not capable of performing housing activities on her own, and every time she needs help she calls one of her sons. Maria's primary concern is her angina pectoris that requires her to take medication on a daily basis. Lately she has been feeling some memory problems and is afraid she will forget to take her medication. For these reasons she is not satisfied with her current health condition. Maria's biggest fears are forgetting to close doors or windows or take her medications.

It is clear that this persona needs a distinct solution from what is available to a common person, and the services provided should be aimed primarily to the persona's health condition. Therefore, the presented Scenario 1 must be adapted to respond to the specificities of Persona 1. The hardware can be maintained, and as stated before, can be used to attend to different maximization objective. But the services and actions logic must be modified, as in this case even the concept of comfort is different from user to user; thus, personalization is key in these systems.

While AmI is very much focused on the *home*, the environment in AAL is wherever the user is located, which broadens the range to users walking outside on the street or being monitored while at work. This type of monitoring concept is associated to Body Area Networks (BANs) which consist of a set of sensors (very often biosensors for reading vital signs), and a transmitter that allows the user to move around without being dependent on home sensor systems [27–34]. There are still some issues related to the privacy of the users, and this is currently being debated in the UE. The user private sphere and the classification of medical information are the main themes of this debate [35].

Hardware miniaturization enables increased mobility, while increased computing power enables fast data processing, but human supervision of incoming data is still required, which burdens several people with the task of reviewing massive amounts of information. Although the final user observes only the benefits, this situation essentially shifts work from one place to another. Therefore, providing greater intelligence to AAL platforms is imperative and can be considered a work in progress.

This paper is organized as follows: in Section 2 the Artificial Intelligence (AI) approach towards the AAL concept is presented, through a detailed architecture along with the challenges of this approach. In Section 3, state of the art projects and solutions that follow the architecture presented in the Section 2 are reviewed. In Section 4, the UserAccess and the AAL4ALL projects are described, including the architecture, implementation, and tests of the UserAccess development. Finally, in Section 5, conclusions are drawn, providing an overview of the relevant areas of the paper and the current UserAccess development state.

## 2. AI in the AAL Context

**Figure 2.**
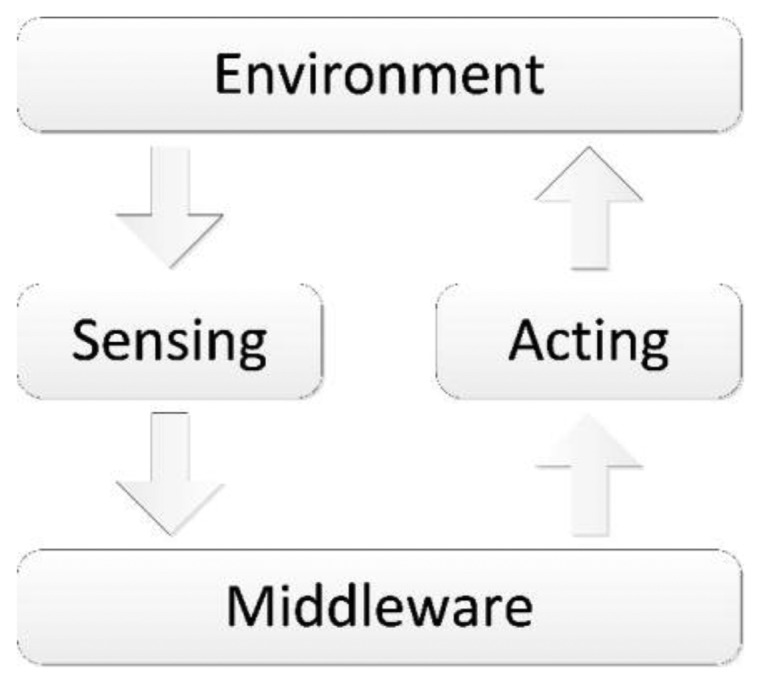
Information cycle of the AAL concept.

As stated before, the AAL at its essence possesses the capability to promote security and comfort, providing an integrated solution that connects several distinct electronic devices to form a unique solution [36,37]. However, these features are not enough by themselves. The aim is to accommodate people, and people change. Not only are our tastes different today than they were yesterday, but our health state today may be different tomorrow and likely also different from yesterday. Thus, it is only reasonable to expect that platforms built to directly care and assist people will adapt and evolve as those people change. A major issue is that, until very recently, AAL solutions overlooked this fact and produced systems that attempt to be—and are undoubtedly—useful, but which are also strict and highly inflexible to change. The information cycle is displayed in Figure 2, where the data received from sensors is passed to the pre-programmed middleware, leading to the response commands being sent to the actuators that change the environment state. Moreover, each person is unique, demanding a personalized solution. Additionally, inflexibility in these systems implies that, for each person, a technician must be assigned to configure the system preferences, and may have to carry on multiple periodic adjustments to each person's individual system. This leads to an endless backlog of interventions, and cannot be scaled up.

**Figure 3.**
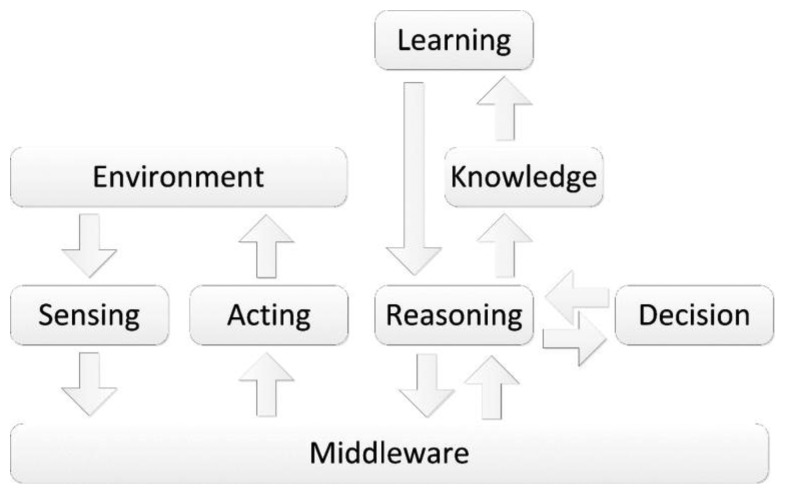
Information cycle and decision process of the AAL concept, resourcing to AI.

Our way to tackle this problem was to introduce the ability for the system to learn from the interactions with its users, resorting to the Artificial Intelligence (AI) domain. Using Figure 2 as a starting point, the difference from introducing AI over the initial concept is illustrated in the Figure 3. Unlike before, the flow of the information is quite distinct, with the middleware becoming the common link between the physical and the logical processes [38]. Therefore, the process is as follows:

1. Sensors send data to the middleware, which is responsible for transforming raw data into low-level information so it can be consumed by the logical framework. This process is required due to the heterogeneity of the available sensors;
2. The middleware sends the low-level information to the logical framework, which then sorts the information according to its type and priority;
3. It is then verified whether any previous action learned is similar to the incoming one, and if so, the same response is provided. If it is a new action, the root strategy will be accessed to clarify the attribute of the sensors involved and the action will be broken down to be reasoned by parts. Similar cases to the parts will be fetched and a response will be constructed from the most similar cases;
4. The framework will then acquire this decision as learned and send the response to the actuators, scheduling an action monitoring cycle to monitor the user response;
5. In the action monitoring cycle, the system will acknowledge the user response. If there are no changes, the last case remains unchanged, while if there are changes, the case will be updated.

This process leads to a flexible platform, which is not only able to learn from the environment but also to update and modify its knowledge. Furthermore, it can adapt itself to the inhabitants without requiring external influences, thus effectively accompanying them as they change.

Using the previously presented Scenario 1, let us assume that the owner wants the blinds open in the morning, even in the winter. Without having to reconfigure the system, the owner has only to open the blinds whenever he/she wants and after a few occurrences (depending on the logical framework configuration), the system will register this change as a desired action and assume that it should become the standard action, thus starting to open the windows by itself at that time.

Provided with intelligence, the platform will change concomitantly with its user, thus fitting perfectly the AAL aim of enhancing security and comfort but with minimal configuration. It is expected to provide intuitive systems to the elderly and those with cognitive or physical impairments, since no better system can be expected than one not requiring any more interactions from users than their typical actions on the objects that they already use. Furthermore, unexpected but beneficial actions and knowledge, beyond those that could have been purposefully engineered into a traditional system, may result in this framework. In fact, this can lead to emergent behaviour, a common feature in complex systems, such as the system implementing an action that the user is repeatedly making without even being aware of it. If the user had been asked to name a list of features to be programmed onto a static platform, he would certainly leave out some potentially useful ones, and also that system would be unable to identify and learn them.

Another outcome of this AAL concept was the Body Area Assisted Environment (BAAE), which is an extension of the BAN. While the BAN only monitors the user without providing any interaction, where in most cases the generated information has to be sent to other devices and analysed by specialists, the BAAE generates a sphere of knowledge, resorting to the latest technological devices to monitor and interact with the user. The more recent smartphones can provide mobility and computing power, act as communication platform and human interfaces, and even include some sensors (e.g., GPS, gyroscopes, luminescence, sound, *etc.*), while wearable sensor systems can provide specialised data about the user's health condition. The combination of these may provide pertinent information not only to the user, but also to physicians or relatives tasked with caring for that user.

The Person-Centric Computing area has advanced projects that can be used on the architecture phase, outlining the human-computer interfaces design, aiming towards an intuitive and unobtrusive operative environment [39–43]. Although some defend the principle that the user will be overwhelmed by using a large set of technological devices, we do not share that point of view. In fact, we believe it is quite the opposite, and the problem resides not on the quantity but on how they are used [44–47]. To illustrate this point we can take smartphones or tablets as an example. The lead selling advanced devices [48,49] have various features, such as phone call ability, Wi-Fi, full web access, video/audio reproduction, rich visual interfaces, vibrating feedback and sensor systems, and for instance if a user wants the directions to a geographic location all they have to do is open the GPS application and type the address. This example clearly demonstrates that the simplicity must rely in the “usage” process [50]. As we emphasize before, the integration process must be used to “hide” the devices from the user, leaving only a unified user interface, thus using simple ways to perform complex tasks.

### Challenges

Adaptation to the environments that surround the user is crucial for the operation of these platforms. The problem with AALs and even with BAAEs is that they can be very diverse, thus generating disperse or even noisy data. This type of data will confuse the reasoning process and may provide different results from those expected. This can lead to major problems and is the main reason why there are so few projects based on mobile environments. The challenge lies in the concept of environment.

The environment is established as a space that possesses objects at approximately the same location at all times, with the same being true of sensors and actuators. The reason for this is that calculations and verification of assumptions are considerably easier to perform if the placement is near-static. For instance, a camera placed at a corner of a room has a dissimilar perspective of one placed at the middle of the room, and one that would shift location to that extent would provide images that would be extremely difficult to have a reliable prediction or reference to match against.

Another challenge is the real perception of the environment. Even if a room is equipped with a large set of sensors, their combined information will differ greatly from a human perspective. The context obtained is “machine-like”, being populated to the maximum ability of the sensors, but still perceiving only absolute information. An illustrative example is a person navigating with eyes closed within their own house. Surely, some bumps with furniture and objects are expected to occur, but having a memory-imprinted map allows a fairly decent capability to know if the person is in the hallway or in the living room, for example. Thus, one has to realize that the platform only has knowledge of what it quantitatively perceives and is unable to reason about information that was not obtained by the sensors (or perform some of the complex mental operations that we intuitively apply on a daily basis).

To master these challenges, and still some others, recent projects are presenting novel approaches in several domains where the results can be used in AAL platforms.

## 3. State of the Art

Presently, there are projects that propose new approaches in the AAL domain. Some have direct impact on final users, while others (typically more specific) are focused on providing major evolution in undeveloped areas of the AAL.

In terms of guidance systems there are two types of spaces: indoor and outdoor. In the case of indoor systems, an augmented reality guidance system is presented in [51], which promotes mobility and allows people with cognitive problems to be located in real-time. It uses a smartphone with a camera and presents the user with a direct video feed having overlapping direction arrows to indicate the route. Moreover, this project features a web and mobile platform for the user's caregiver, allowing real-time route verification and editing, and supporting multiple users. This alleviates the caregiver's burden and enables multiple-user monitoring. A novelty is the introduction of allowed areas, associated with a warning if the user leaves a certain area or travels a great distance. A comparable project is presented in [52] with the difference that it uses landmarks pictures to guide the user, thus enhancing the user's visual memory. This project relies on predefined paths that the user is accustomed to use.

In terms of indoor location, a plethora of different works are currently under development. A ZigBee-supported user location system is presented in [53], supporting multi-user location simultaneously. It resorts to a high number of devices to perform triangulation, having a complex architecture. The alternative use of Wi-Fi networks to detect the user is proposed in [54], using a Wi-Fi tag (such as a smartphone). This project takes advantage of several computation processes, such as the time of arrival, received signal strength and angle of arrival, to calculate the distance to several base stations (like household Wi-Fi routers). This concept is supported by the increasingly available home networks and smartphones, sparing the acquisition of additional devices than the users already have. An analogous work is proposed in [55], where ultra-wideband radio frequencies are used to locate the user. The location process is similar to that of [54], although it uses proprietary hardware to bypass the saturation problem that can occur using Wi-Fi systems. Finally, the LAURA system is presented in [56], which allows locating and tracking users in a nursing home, using ZigBee wireless networks. This system also monitors the users resorting to accelerometers to detect sudden movements and to increase the accuracy of the location detection.

Martínez-Martín *et al.* [57,58] presented a real-time visual system, which detects, recognizes and tracks people and target objects. This system, despite being mainly aimed at robotic tasks, can be deployed in an AAL environment. The novelty of this project is its possibility of distinguishing between several objects without any knowledge about the environment and any special environmental conditions. Thus, it can provide effective tracking properties, perfectly suitable to locate objects in an individual's home, which can be an important feature for an AAL platform. Also, in the visual detection systems area, the work described in [59,60] offers a heterogeneous platform that is able to detect several people in a space and track them freely. This platform is implemented in the form of a multi-agent system, thus able to connect to any platform (given the proper ontologies), a useful feature for AAL. Moreover, it is able to distinguish unique individuals in a crowded place.

A crucial feature that has gathered much attention in AAL platforms is fall detection, since these systems are aimed at the elderly population [61]. In [62] an initial attempt is presented to structure the human body movement, listing the several types of movement and how to interpret them computationally. The high detection accuracy (97%), made this work a cornerstone, which has since spurred a myriad of subsequent research. The following movements were detected: Walking, standing, sitting, lying down, sitting to standing, standing to sitting, bending up/down, lying from sitting, and sitting from lying. Accelerometers and gyroscopes are used to gather information on the direction and the force involved in each movement, allowing it to be identified and classified. Gjoreski *et al.* [63] presented a system composed of three wireless accelerometers placed on an individual's body for posture recognition. Using an elimination process and a force value threshold, it is able to detect whether a movement is outside a predefined range, in which case a warning is activated and sent to the base receiver to be processed. To enhance the results provided by the latter work, one could combine also the work published in [64], which consists in wearable wrist bracelets that determine the task that the wearer is performing. Tasks tend to be repetitive, and the way humans perform them is also repetitive; thus, once the system has learned the way a user performs a task, it is able to detect (by comparison) what the user is doing.

## 4. UserAccess Role in the AAL4ALL Project

AAL4AAL [65] is a Portuguese AAL project consisting of a consortium of 31 partners, that aims to improve the life of the elderly by establishing a framework that will enable and foster the adoption of technological devices and services. The goal is to create an ecosystem of services and devices certified according to Portuguese legal (and medical) regulations. One of the main advantages of this project is allowing remote monitoring by informal or formal caretakers. The informal caretakers can be relatives, friends or anyone in the user's acquaintance circle, whereas the formal caretakers can be doctors, nurses, or specialized technicians. The task of both groups is to care for the user, providing assistance when needed but without having to be physically present at all times. This project can support people who carry mild cognitive impairments and mild to severe physical disabilities, enhancing their independence, by decreasing the need of constant supervision by other people.

By adopting the idea of integration in this project, the inclusion of new devices and services by other developers (outside the consortium) is promoted, contrary to most other projects that allow only a set of partners to deploy products. This possibility is achieved through the means of certification; the project foresees and is already implementing a certification procedure that establishes operational rules, supporting a business logic that suits the AAL4ALL ecosystem, and could be adopted on a national level for the future development of new services and devices by any company (which can then submit them for certification).

The main goal is to provide a system that an individual can simply buy in a store, take it home, and by pushing a button it becomes automatically setup with the rest of the environment (both physical and service wise). As an example, if a user buys a smart weight scale with an AAL4ALL certification, upon turning it on at his home, the scale should be able to connect to the home platform and publish the data on the user's health channel and eventually updating the user's medical profile. This would make that information immediately available to caregivers.

This project also proposes an open market for caregiver companies, since the business logic and communications plan are openly available. Anyone that obtains a government issued certification can immediately start providing caregiving services taking advantage of the established framework.

The implicit heterogeneity of the AAL4ALL project solutions implies that each partner is responsible for the development of a component of the overall project. In Section 4.1 we present a specific AAL4ALL solution case-study, the UserAccess project, one of the many different solutions developed within the consortium.

### 4.1. UserAccess

Following a user-caregiver connection, we have devised a service that allows the caregiver to directly monitor a user, or several users. UserAcess [37,66] is a mobile and web project that fetches data from an AAL4ALL Node and presents it in a human-readable way.

The AAL4ALL Node is an information bus gatherer that receives, stores, and sends information about the user. It consists on a cloud modular server with REST connection abilities that serves as a collective information gateway for everything that is connected to the platform, and thus possesses information about all users. Access is granted through user and password tokens that secure the appropriate data channel, assuring the privacy of the platform users and directing the information of a specific user only to the appropriately subscribed caregiver(s). A problem arises due to the fact that this implies a large amount of data, and most of it is not easy to interpret, something which usually would force the caregiver to read and interpret extensive information, defeating the purpose of the project. The UserAccess solution was devised to consume the information on the AAL4ALL Node, and locally process that data in order to transform it into information about the user, and then publishing that information to the responsible caregiver.

**Figure 4.**
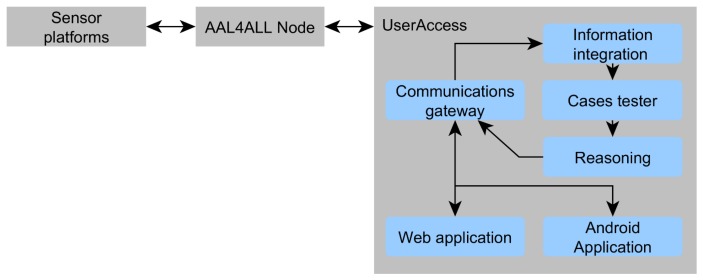
UserAccess architectural components.

**Figure 5.**
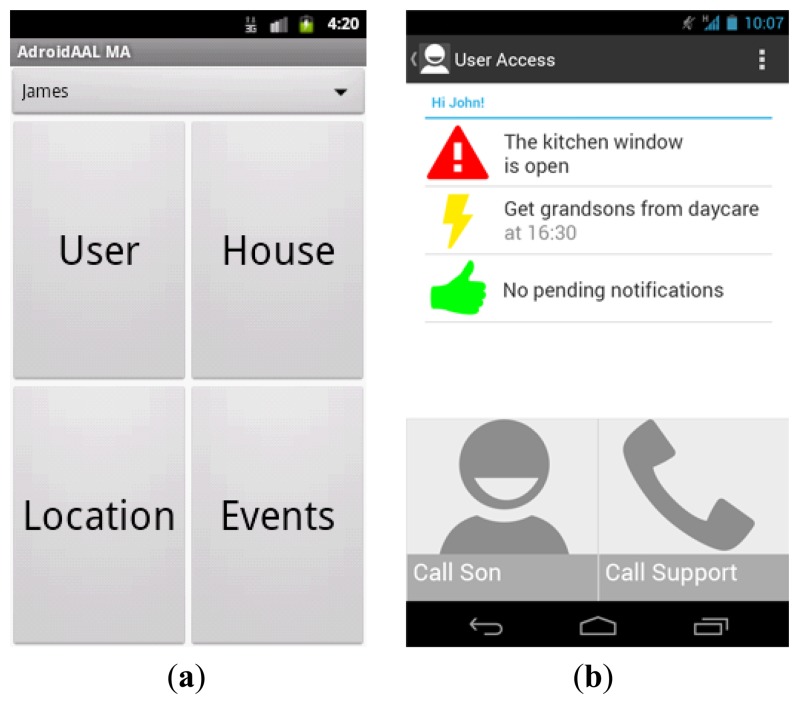
(**a**) The Android application home interface. (**b**) The user's warnings and activities, allowing the caregiver to call the user's son or tech support.

Illustrated in Figure 4 is the UserAccess platform: it connects to the AAL4ALL Node, which in turn is connected to the *sensor platforms* (wherever they are located). The Node enforces the usage of high-level messages so that data is easily consumable and coherent for all services. In UserAcces, the following simplified structures are present:

- Communications gateway: the entry and exit point of all communications. It consists on a Tomcat apache server with the REST communications protocol, implemented in a multi-agent system (MAS). The MAS assures that any modification or new feature can be easily deployed. Moreover, the *Communication gateway* assures the communication tunnel between the web application and the Android application;
- Information integration: assures the conversion of data in the Node for UserAccess internal consumption. The logic processing implemented in the Cases tester and the Reasoning require that the information be filtered and translated. Moreover, the information integration has in its architecture a pre-processing module that is able to fuse some of the data received, according to the type of defined sensors;
- Cases tester: Implements a rapid analysis in search of cases similar to incoming information. Using the clinical guidelines [67] concept, the goal is to implement a filter system that has pre-determined rules on which it is possible to act fast and directly. For instance, if there is a sudden drop on the value reported by an EGC sensor, this module will generate a warning and send it directly to the caregivers. By establishing some rules (mostly health related, in large part because of the response times typically have to be very short), the system can act rapidly to some critical events;
- Reasoning: responsible for the actions taken by the system. This module resorts to logic in order to reason about the occurred situation. If the Cases tester is unable to resolve the event, the Reasoning will receive the most similar cases and opt for one, saving this decision for subsequent occurrences. Furthermore, it will append a revision flag to be reviewed in the future. Last, it considers user actions in order to build a user profile that can contribute to better adjust the information the caregiver receives;
- Web application: A web page that displays basic information about the user being monitored, thus allowing status inquiries from anywhere. At the current stage it does not possess any bidirectional communication, and only allows access to information;
- Android application: has the most advanced user interface of the UserAccess. It is built according to usability guidelines, presenting succinct information about the monitored user and having simple and intuitive buttons that require less than three interactions to obtain the information. The caregiver interface is shown in Figure 5, namely the home interface, with intuitive and straightforward buttons, and the personal user interface, with pending warnings and the user's activities.

Also illustrated in Figure 4 is the flow of the information. The UserAccess platform periodically consumes the information present in the AAL4ALL Node, using the *Communications gateway*. Then, information is treated in the *Information integration*, and sent to the *Cases tester*. If the latter has an identical case in storage, it queries the *Reasoning* for an answer and publishes it in the UserAccess data stream. Otherwise, it sends the most similar cases to the *Reasoning* to decide if there is any warning or anomaly that should be notified to the caregiver. The data stream is always available via web application, while the Android application will verify the data stream periodically, to minimize power consumption and optimize battery usage and allow a smooth operation of the other applications.

The implementation of the server modules is based on the MAS concept, being highly modular and following a web service type of communications that guarantee integration of other developers' modules. For each sensor or sensor platform, a data interpretation guideline must exist in a module format, requiring the upload of that guideline to the platform by the hardware producers.

### 4.2. Case-Study

In order to validate its behavior and performance, this platform was tested in a controlled environment with the following set of ZigBee sensors:

- One base station;
- Two movement detection sensors;
- One open/closed door sensor;
- One light sensor;
- One temperature sensor;
- One touch sensor;
- One AC current on/off switcher.

The base station was connected to a laptop acting as the middleware and publishing in an intermediate server, acting as the AAL4ALL Node. After a training session, the cases were uploaded to the platform and trial tests were conducted. The sensor platform was developed within the AAL4ALL project by another development team from the University of Minho, and due the project development phases, this sensor platform was the only one used. Additionally, the GPS sensor directly available on the user's smartphone was used.

#### 4.2.1. Sensor Platform Response Time

The sensor platform was tested in terms of the response time on two factors: network time and stabilization time. The network time is the time difference between the sensed event and the information reaching the server; the stabilization time is the time taken by the middleware and/or the sensor to reach the normal status or to recover from the previous state. These times are important because a critical situation must be reported as soon as possible so the caregiver can act accordingly. The middleware was configured to report to the server only when a sensor changes its status. This decision was made to avoid communication entropy. Also, a wired network was used between the middleware and the server, established at 100 Mbps.

Displayed in Table 1 are the response times (rounded to the second) of the sensor platform. The middleware processing times and the network communication latency are clearly irrelevant, being well under 1 s, thus not creating any limitations in sending data to the server. There are issues on movement detection, open/closed door, and light, having a high stabilization time. For instance, if the user has quickly exited the room, the initial movement detection is quickly and correctly done, but it takes 5 s until the sensor reports no movement. This situation seriously affects the system performance and the correct detection of the occurring situation. Furthermore, it leads to cascading problems, when several sensors are reporting their status and the other sensors are still stabilizing.

Currently, the development team is addressing sensor problems, but the tests were performed with their initial status. The sensor with most problems is the motion detection one, and a different technology is currently being considered to deliver this feature.

#### 4.2.2. UserAccess Procedures

The UserAccess firstly receives the information tagged, in a JSON format, containing information about all the sensors state. The information can assume three states: a numerical/discreet value, “broken” and “@”. The “broken” and “@” are states relating to the lack of faulty information, being “broken” the state when the sensor does not respond in the previous 3 min, and the “@” the state when the middleware has never received information about that sensor. These distinct values have to be normalized, with the real data being separated for further processing.

One of the aim of the development is to provide safety, which was translated into tests where the user exited the house. The following tests were used:

- Test1: the user gets up from a seated position and exits the door;
- Test2: the user gets up from a seated position and open and closes the door;
- Test3: the user enters the house;
- Test4: the user gets close to the door but does not open it.

The other sensors were used to feed the UserAccess with data, in search of hidden correlations between the sensors.

**Figure 6.**
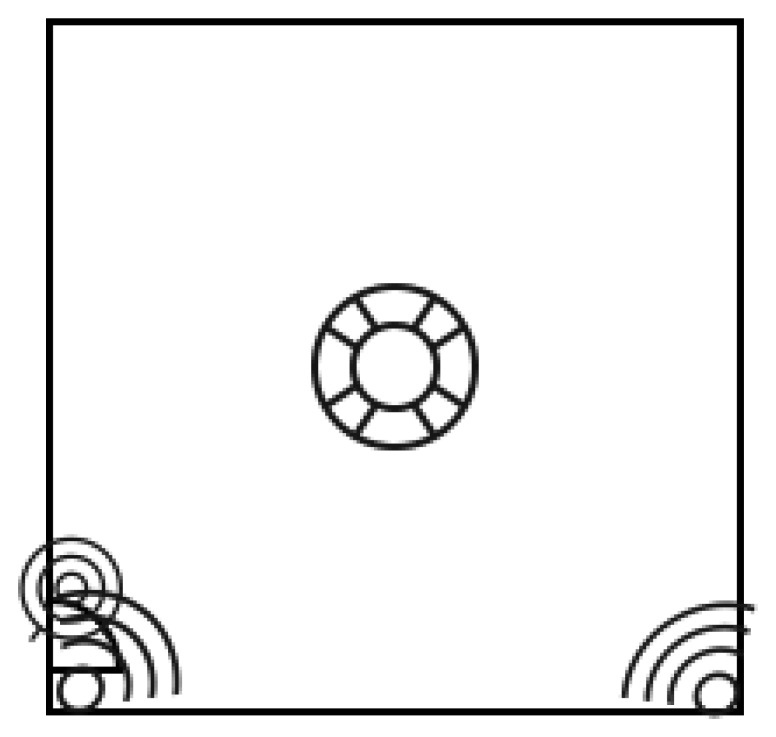
The floor plan of the room. In each corner are the motion sensors and on the door the open/closed sensor. At the center are the rest of the sensors and the middleware.

The area used was a laboratory room with the dimensions of 7 m width by 7 m length by 3 m height, and the sensors were placed in the door area, minus the environment sensors, which were spread across the room. This sensor placement maximized the intentions of the planned tests, as shown in Figure 6.

After a sequential execution of all tests 30 times, the data was processed to find underlining relations. The WEKA tool was used to obtain the data classification and association. For the classification procedure, the J48 classifier was used, where the outcome is a relational tree. This procedure only revealed that each sensor acts on its own and no sensor is directly related to others, being the values so diverse that the algorithm was unable to produce a usable response. As for the association procedure, the outcome of this test related the two movement sensors with the open/closed door detection; the algorithm used was Apriori, with the distance of 1 and the maximum rules of 10. Although it is quite trivial to achieve this conclusion, it is our perspective that this association was obtained because of the stabilization times of the motion sensors.

From the previous observations and the results of the associations obtained from the tests, the following associative logic was devised:

ϑ = {*sensorStates*}
ε = {*previousAbsoluteStates*}
Δ = {*event*(0,0)@*sensorStates, event*(0,1)@*sensorStates*, …, *event*(*n*, *m*)@*sensorStates*} Λ *n* ∈ {*availableSensors*} Λ *m* ∈ {*possibleStates*}
*α* = {*association, registerError*}
*℘ is the following set of rules*:
*detection*(*A, B*) ←.
*detection*(*A, B*)
← *previousStates*(*A, B*), *event*(*A, F*)@ *sensorStates*, *associated* (*F*), *detection*(*F, B*).
*detection*(*A, B*)
← *previousStates*(*A, B*), *event*(*A, F*)@ *sensorStates*, ∼*association*(*F*), *detection*(*F, B*).
*previousStates*(*A, B*) ← *event*(*A, F*), *previousStates*(*F, B*).
*eventa*(*X, Y*) ←. Λ *X* ∈ {*availableSensors*} Λ *Y* ∈ {*possibleValues*}
*τ is the following set of integrity constraint*:
⊥ ← *registerError*(*F*), *association*(*F*)

These set of rules establish that the previous state always influences the next state, meaning that to infer the context and the action that the user is performing, the system has to know the absolute previous state. The absolute previous state is the previous context state which incorporates all of the system's sensors. This way, a subset of context is obtained, creating an association between all sensors, with the map being a weighted graph. Furthermore, the sensors states are considered separately in the Δ as they shift the association values; thus, the current state is influenced by a combination of the current sensor value and the previous state. Finally, due to the previously explained issue where the middleware could report sensor problems, the *τ* assures that those errors do not influence the association values or the current state mapping.

**Figure 7.**
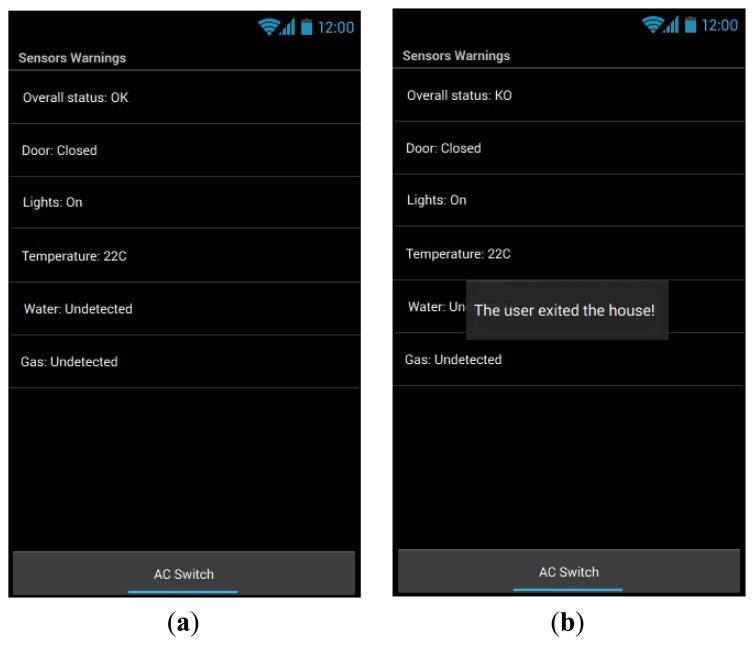
(**a**) The visual state representing the stabilized context “initial” which means that the user is out of the sensors reach and the last five previous states are equal. (**b**) The user has performed the action of “leaving the house” which involves the motion sensors and door sensor.

Resorting to the findings in the association procedure and the associative logic, the motion sensors and the door sensor were associated and conditions relating them were implemented on the UserAccess *reasoning* module and reflected on the *cases tester*. The visual interface was configured to display the context actions and control the AC switcher. In Figure 7 is represented the initial sensor detection state and on the right side the warning presented when the user has exited the house.

The distribution of the tests were as follows:

- Ten executions of Test1
- Ten executions of Test3
- Five executions of Test2
- Five executions of Test4

These distributions were planned to ensure that the basic actions were correctly identified, and thus the warning was displayed. The heterogeneity and the plug-and-play features of the sensors are greatly considered, and although the information from the middleware is high-level, which aids the logic reasoning, our aim is to identify the environment and actions context with any sensor available.

The tests results can be seen in Table 2, where the positive detection is shown for all batch of tests. These represent the acuity of the tests and the UserAccess correct detection of the environmental context.

In these tests, the system accounted for the sensors' stabilization times, and if after a period of time the sensor would not be stabilized the system assumed that other action was taking place, and thus the system reported a negative detection. The presented data is still far from what was expected, which was a 90% positive detection in each test, being the reason the hardware and learning mechanisms conditions.

## 5. Conclusions

AAL projects are rapidly innovating and advancing towards increasingly complex systems, requiring unified solutions that have to resort to methods other than traditional inflexible middleware. As such, considerable benefit can arise from the application of AI to AAL, providing the latter with self-learning procedures, enabling the platforms to evolve along with the users, and avoiding the need for (repeated) external adjustments.

The AAL4ALL project is a beacon in the AAL area, presenting a novel architecture that supports the creation of an open ecosystem able to absorb the most modern advances in hardware and software, and ensure the needed integration processes between them. Moreover, by being structured around a certification entity, the AAL4ALL project will provide a much needed breakthrough in terms of unifying current and upcoming AAL projects, facilitating future collaborations.

The UserAccess project was created as an AAL4ALL product and a test case for the feasibility of the entire AAL4ALL project concept. At the current state of development of the UserAcess platform, periodic tests have shown good results, thus validating the approach. The *reasoning* module is a challenging task to implement and is currently undergoing improvements, using a case-based reasoning approach and constrained Bayesian Networks. The interfaces are stabilized and due to the MAS nature of the project, we are able to run experiments during the development phase while presenting the information as it would be perceived by a caretaker.

Finally, the UserAccess progress thus far has proven that it can be a standalone AAL project, exhibiting novel features, such as the integrated sensors-human interaction and an open platform. It focuses on an underdeveloped AAL area which is caregiver assistance. The caregiver plays a vital role in the user's life and will significantly determine the user's wellbeing and quality of life.

## Figures and Tables

**Table 1.** Sensor platform response times (the values were rounded to the second) after 30 actions.

**Sensor**	**Stabilization Time**	**Network Time**
Movement detection	5 s	<0.1 s
Open/closed door	0.5 s	<0.1 s
Light	1 s	<0.1 s
Temperature	<0.1 s	<0.1 s
Touch	<0.1 s	<0.1 s
AC current on/off switcher	<0.1 s	<0.1 s

**Table 2.** The tests batch and the positive detection of each test.

**Tests**	**Positive Detection**
Test1	7
Test2	1
Test3	5
Test4	2
